# The Isolation of Hypoglycaemic Compounds from *Desmodium canum* and Their Synergistic Effect on Blood Glucose Levels in Euglycaemic Sprague Dawley Rats

**DOI:** 10.1155/2020/9465139

**Published:** 2020-10-19

**Authors:** Kemmoy Lattibeaudiere, Roy Porter, Ruby Lisa Alexander-Lindo

**Affiliations:** ^1^Department of Basic Medical Sciences, Biochemistry Section, The University of the West Indies-Mona, Kingston, Jamaica; ^2^Department of Chemistry, The University of the West Indies-Mona, Kingston, Jamaica

## Abstract

*Desmodium canum* (Strong back) commonly consumed as a tea or tonic is believed to possess hypoglycaemic activity. This paper sets out to isolate potential hypoglycaemic compounds present within the plant and investigate their synergistic effects on blood glucose levels in euglycaemic Sprague Dawley rats. The milled plant was sequentially extracted using hexane, ethyl acetate and methanol. The ethyl acetate extract was subjected to column chromatography yielding seven major fractions and were subsequently bioassayed using the Oral Glucose Tolerance Test (OGTT). Further chromatographic separation and analysis using Gas Chromatography–Mass Spectroscopy and Fourier Transform Infrared Spectroscopy enabled the identification of two hypoglycaemic compounds, oleic acid (OA) and succinic acid (SA). These were bioassayed individually and as a cocktail to determine the synergistic effects using OGTT. Intravenous administration of these compounds individually indicated both are very potent in retarding blood glucose levels. However, the most significant activity was observed on synergistic administration. The cocktail (1 : 1) displayed significant hypoglycaemic activity throughout the entire study. It also significantly differed from OA at the 120 min interval (3.43 ± 0.22 mmol/L vs. 4.98 ± 0.19 mmol/L, resp., *p*=4.29 × 10^−7^) and significantly differed from SA at 30 min (3.95 ± 0.43 mmol/L vs. 5.17 ± 0.32 mmol/L, resp., *p*=0.003), 90 min (4.35 ± 0.36 mmol/L vs. 5.49 ± 0.69 mmol/L, resp., *p*=0.04), and 120 min intervals (3.43 ± 0.22 mmol/L vs. 4.94 ± 0.31, resp., *p*=1.54 × 10^−5^). Oral administration of the cocktail showed comparable potency to that of metformin (*p* > 0.05) throughout the OGTT curve. The synergistic effects of the naturally isolated compounds yielded higher potency levels than individual administration and when administered orally, the hypoglycaemic effect was similar to that of metformin. This may assist in paving a way to attempt a novel method in approaching antidiabetic therapy.

## 1. Introduction

Diabetes mellitus (DM) is a group of metabolic diseases that is characterized by hyperglycaemia resulting from defects in insulin secretion from the beta cells, insulin's action, or a combination of both [1]. The most predominant form of diabetes, type 2 diabetes mellitus, affects millions of persons worldwide and is typically as a result of deficiency of insulin action in peripheral tissues. This results in an inability of translocation of the glucose transporter 4 (GLUT4) proteins in these tissues and thereby preventing uptake of glucose from the blood [2].

The pathophysiology of the disease stems from hyperglycaemia and can result in a wide array of complications that may range from polyuria to cardiovascular complications, erectile dysfunction in men, among others [3, 4]. There is generally a correlation between the complications and the cost associated with diabetes. In low- and middle-income countries, many diabetic patients have turned to traditional medicine to combat hyperglycaemia [5]. Traditional medicine, commonly called folklore medicine, has a long history and existed as the sole source of medicine in the past. It is used in the maintenance of health, as well as in the prevention, diagnosis, and treatment of physical and mental illnesses, where herbs, animal parts, and minerals are employed to be the source of medicine [6, 7]. In modern societies, there has been a general decline in the use of folklore medicine [8]. On the contrary, many developing societies still rely on the use of traditional medicine as the primary source of treatment. With this information, many scientific journeys have been embarked on, in an attempt to discover novel pharmaceutical agents. To date, a large amount of research on plants has led to the extraction of phytochemicals with hypoglycaemic properties. For example, the traditional plants, Flinders rose (*Capparis spinosa*) [9], Garlic (*Allium sativum*) [10], and *Pilea elizabethae* [11] are a few of the plants that have been studied and shown to have hypoglycaemic activities.

In the Caribbean, *Desmodium canum* commonly called Strong Back is consumed as a tea or tonic for its potential ability to alleviate the symptoms of diabetes, asthma, the common cold, urinary problems, and erectile dysfunction [12, 13]. As a member of the Fabaceae family, the plant exists as a herb that grows up to about 70 cm. It is found in many Caribbean territories where it plays a significant role in herbal medicine [14]. With the knowledge of the uses of many of these plants, research into the active pharmacological agents has stemmed the drive to discover and supply alternative and more efficient types of treatment to the local research industry [13, 15, 16]. The discovery of plants with hypoglycaemic and antidiabetic capabilities is an ongoing process and will never truly be exhausted. However, it is crucial that significant breakthroughs are made in the near future due to the rate at which the number of persons affected by diabetes mellitus and its complications is increasing with time. Alleviating or more effectively treating anything that affects health, family, and the society at large is of importance to all individuals [17], hence the need to embark on this research journey to isolate hypoglycaemic compounds present in *Desmodium canum* using various chromatographic techniques and to assess their synergistic effect on blood glucose levels in normoglycaemic Sprague Dawley rats.

Various techniques in chemical analysis are widely used in qualitative and quantitative analysis of natural products. Some of the most widely used include Gas Chromatography–Mass Spectrometry (GC-MS), Fourier Transform Infrared Radiation (FTIR), and carbon-13 and proton Nucleic Magnetic Resonance Spectrometry (NMR) [18]. Together, these techniques form a powerful tool in analyzing and elucidating the structures of natural products.

## 2. Materials and Methods

### 2.1. General Experimental Procedure

Hexane, ethyl acetate, dichloromethane, acetone, and methanol (HPLC or ACS grade) were purchased from Pharmco Products (USA) and were used for extraction and purification of phytochemicals. Dimethyl sulfoxide (DMSO) and Tween 20 were purchased from Sigma-Aldrich (USA) and served as vehicle and negative control. ACCU-Check Active Blood Glucose Monitoring System purchased from Lasco Pharmaceuticals was used to monitor blood glucose concentrations; Analtech silica gel grade 12, 28–200 mesh, was used in column chromatography; and precoated F254 Si 60 glass plates of 250 *μ*m thickness from Sigma-Aldrich, succinic acid, and oleic acid BioXtra >99% standards were purchased from Sigma.

#### 2.1.1. Plant Collection and Extraction

The plant was collected between the months of January and March in the hills of rural St. Andrew, Jamaica, and was authenticated (voucher number: 36277) by Mr. Patrick Lewis, Herbarium Department of Life Sciences, The University of the West Indies (UWI), Mona. The stem and the leaves were air-dried and milled into a fine grounded powder using an electrical grinder. The plant extraction was done similar to the protocol described by Alexander-Lindo et al. [19] and Vanisree et al. [20]. The dried, milled aerial plant material was extracted sequentially using hexane, ethyl acetate, and methanol at room temperature. This was done for two separate 8 h periods followed by a 24 h for each solvent. The solvents were removed at reduced pressure (80 mbar) using a rotary evaporator. This yielded three separate crude extracts; hexane extract (yield: 0.76%), ethyl acetate extract (yield: 0.48%), and methanol extract (yield: 2.34%). The ethyl acetate extract was investigated for its hypoglycaemic potential while the hexane and methanol extracts were used for other assays outside the scope of this paper.

#### 2.1.2. Purification of Ethyl Acetate Extract

The biodirected purification of the crude ethyl acetate hypoglycaemic extract of *Desmodium canum* was achieved through an alternative Oral Glucose Tolerance Test (OGTT) and chromatographic method.

The crude ethyl acetate extract (14.32 g) was subjected to flash column chromatography using silica gel grade 12, 28–200 mesh, with a gradient elution of hexane: ethyl acetate solvent system with increasing polarity (from 100% hexane to 100% ethyl acetate). A total of 128 fractions (30 mL each) were obtained and pooled into 7 major fractions, KFL1 to KLF7, based on similar TLC (silica particle size: 5–17 *μ*m, fluorescent indicator; thickness: 200 *μ*m) profile. KLF1 existed as a light green gel-like solid (yield 4.3%, 615.8 mg), KLF2 was a reddish-brown sticky gel (yield 3.5%, 501.2 mg), KLF3 appeared as a very thick, light brown waxy solid (yield of 6.3%, 9021.6 mg), KLF4 was constituted of clumps of light brown solids (yield of 4.8%, 687.4 mg), KLF5 on the other hand was a yellow gel-like solid (yield of 11.9%, 1704.1 mg), KLF6 was a light green gel-like solid (yield of 17.7%, 2534.6 mg), and KLF7 was a very dark solid, similar in appearance to the crude ethyl acetate extract (50.8%, 7274.54 mg).

**Figure 1:**
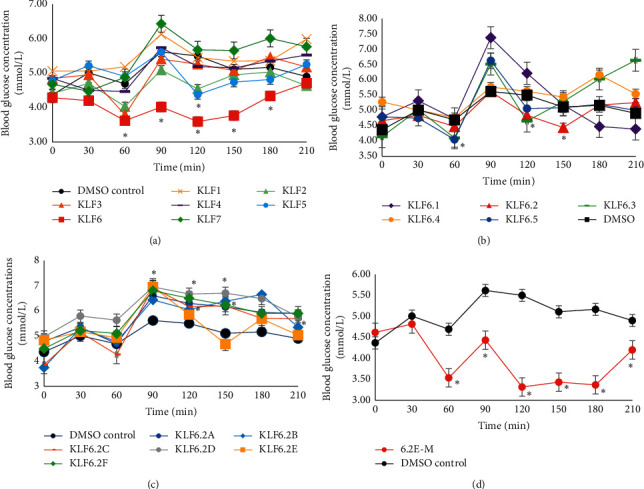
OGTT of fractions obtained from EtOAc extract of *Desmodium canum* compared with DMSO control. (a) Fractions KLF1–KLF7 (intravenously administered at 40 mg/kg BW). (b) Fractions KLF6.1A–KLF6.5 (intravenously administered at 25 mg/kg BW). (c) Fractions KLF6.2A–KLF6.2F (intravenously administered at 15 mg/kg BW). (d) Fraction KLF6.2E–M (intravenously administered at 15 mg/kg BW). Data are expressed as mean ± standard error of mean, with ^*∗*^ indicating statistical significance when compared with DMSO control (*p* < 0.05).

The fractions were then bioassayed using the OGTT which showed that KLF6 had the most hypoglycaemic activity (Figure 1(a)). The lime-green gel-like KLF6 (2.001 g) was chromatographed using a solvent mixture of hexane: acetone and monitored at wavelengths of 254 nm and 366 nm. A total of 71 fractions were collected in 20 mL portions and pooled in five major fractions (KLF6.1 to KLF6.5) based on a similar TLC profile. KLF6.1 was observed as a thick and waxy solid (yield of 8.9%, 178.1 mg), KLF6.2 was a yellow-orange solid with a thin oil layer (yield 34.3%, 686.3 mg), while KLF6.3 existed as an orange gel-like solid with a thin oil film (yield 20.2%, 404.2 mg), KLF6.4 was seen as orange gel-like substance (yield 12%, 240.2 mg), and KLF6.5 was observed as a yellow-brown sticky gel-like substance (yield 11.2%, 224.1 mg). These five fractions were bioassayed using OGTT (Figure 1(b)).

KLF6.2 showed the most significant hypoglycaemic activity and was further separated (∼600 mg) using gravity column chromatography with dichloromethane (DCM): acetone mixture with increasing polarity (from 100% dichloromethane to 100% acetone). One hundred and twenty individual fractions (5 mL) were collected and grouped into six major fractions based on a similar TLC profile (KL6.2A to KLF6.2F). KLF6.2A was a yellow wax with the presence of tiny crystals (yield 0.86%, 5.16 mg), KLF6.2B was a dark green solid with very dark green precipitate (yield 12.3%, 73.8 mg), and KLF6.2C had the highest yield (42.8%, 256.8 mg) and appeared as clumps of dark yellow waxy solid. KLF6.2D was similar in appearance to KLF6.2C but was a lighter yellow wax (yield 22.8%, 136.8 mg) and KLF6.2E was a dirty green solid with white granular crystals (12.29%, 73.74 mg) while KLF6.2F consisted of a light green precipitate with a film of oil (8.3%, 49.8 mg). These were bioassayed to determine which fraction had the highest hypoglycaemic activity.

KLF6.2E showed the most hypoglycaemic activity and was chosen for further analysis. It was noted that KLF6.2E formed crystals on the side of the flask when left standing. The semipurified fraction was washed with cold acetone and filtered using the Buchner apparatus under pressure. This led to the removal of white powdery crystals whose quantity was too low for bioassay. Concentrating the mother liquor by rotary evaporation yielded small greenish clumps with a thin film of oil. This was labelled KLF6.2E-M and shown to have significant hypoglycaemic activity.

#### 2.1.3. Gas Chromatography–Mass Spectrometry Analysis of KLF6.2E-M

KLF6.2E-M was dissolved in HPLC grade chloroform and derivative with N,O-bis(trimethylsilyl)trifluoroacetamide (BTSFA). It was then analysed using Agilent Technologies Gas Chromatography connected to an Agilent MSD 5973N mass spectrometric detector. Samples (1 *μ*L) were injected using splitless mode (injection purge off = 0.75 min) with the following conditions: injector temperature, 250°C; transfer line temperature, 280°C; column, DB-1701, 30 m, 0.25 mm i.d., 0.25 *μ*m film thickness; a ramped temperature program employed starting at 80°C, holding for 2 min and increasing the temperature by 20°C per minute for 10 min and holding for 10 min; and solvent delay, 3 min; carrier gas, helium at a flow rate of 1.2 mL·min^−1^.

Operations were carried out in the Scan mode. The mass spectrum results depicted the presence of several compounds, two of the most abundant being possible succinic acid and oleic acid. This data can be found in the supplementary file, Figure S3. Reference standards of these compounds were purchased from Sigma and analysed under similar conditions. Chromatogram and mass spectrum of this can be found in the supplementary file.

#### 2.1.4. Plate-Liquid Chromatography of KLF6.2E-M

Fraction KLF6.2E-M (∼500 mg) was further purified by preparative thin-layer chromatography (Analtech, silica gel 10 × 20 cm, 250 microns) using hexane: ethyl acetate (3 : 2) as the developing solvent. Four intense bands were observed under ultraviolet light at 254 nm, labelled “Band 1” (*R*_f_: 0.12, yield 43.1%) “Band 2” (*R*_f_: 0.17, yield: 11.2%), “Band 3” (*R*_f_: 0.43, yield: 0.9%), and “Band 4” (*R*_f_: 0.76, yield: 38%). These major bands were compared with the reference standards purchased from Sigma-Aldrich under similar conditions.

#### 2.1.5. Analysis by FTIR

The *R*_f_ values for Bands 2 and 4 were comparable to the reference standards purchased. These two compounds were then anlysed by FTIR spectroscopy.

### 2.2. Ethical Considerations

Ethical approval was granted by the University of the West Indies Ethics Committee for the use of animals in the protocol described below. The approval was assigned number AN 07 15/16 and the documents for this can be found in Supplementary Files 1 and 2.

### 2.3. Oral Glucose Tolerance Test [OGTT]

Seventy-four Sprague Dawley rats with approximate age and weight of 12 weeks and 200 g were used for biological assays. This was constituted of equal numbers of males and females. The animals were divided into groups based on the extracts/compounds being administered, with each group containing 6 rats. Each rat was used twice in the OGTT experiments with their use being separated by a two-week break to allow compounds to be removed from their systems. They were housed in polypropylene cages and were separated based on their gender as well as the extract being treated with. The room was maintained at standard room temperature and pressure and the animals were exposed to a 12 h light/dark cycle. The rats were fed standard laboratory rat chow and distilled water *ad libitum*.

The OGTT protocol was adopted from a study done by Wilson and Islam, 2012, with minor modifications [21]. Animals were fasted overnight for a period of about 12 h, after which fasting blood glucose readings were taken in duplicate followed by administration of the extracts or vehicle solvent, 10% dimethyl sulfoxide (DMSO), or 10% tween 20 (0.3 mL) which also served as a control. The fractions from the crude ethyl acetate extract were administered at 40 mg/kg body weight (BW) dissolved in 0.3 mL of DMSO. The further semipurified fractions were administered at 25 mg/kg BW or 15 mg/kg BW. Commercially available pure compounds were administered at 30 mg/kg BW and the cocktail of compounds was administered in a 1 : 1 ratio at 30 mg/kg BW. For oral administration, the cocktail was administered at 800 mg/kg BW dissolved in 0.3 mL of 10% Tween 20. Subsequent to administration, blood glucose readings were taken at 30 min intervals for 1 h, followed by an oral administration of glucose at 1.75 g/kg BW. Blood glucose readings were then taken at 30 min intervals for a further 2.5 h and comparisons were made between the treatment groups and the control.

At the end of the experiment, the animals were sacrificed via intraperitoneal injections of sodium pentobarbital at a dose of 65 mg/kg body weight.

### 2.4. Statistical Analysis

Results were expressed as mean ± standard error of mean. Data were analysed using Student's *t*-test where values were compared to negative and positive control. Values of *p* < 0.05 were considered to be statistically significantly different from controls.

## 3. Results and Discussion

### 3.1. Bioassay of KLF1-KLF7

Column chromatography of the ethyl acetate extract yielded 7 major fractions, of which KLF6 showed the most hypoglycaemic activity when compared with the DMSO control. In the fasting region of the curve, KLF6 caused hypoglycaemic activity of 3.62 ± 0.37 mmol/L versus 4.69 ± 0.26 mmol/L; *p*=0.022 of the DMSO control. At the 90 min interval, KLF6 resulted in a significant retardation of the blood glucose levels. This indicates the presence of hypoglycaemic compounds that prevented a major spike in the blood glucose level (BGL) as seen with the DMSO control (4.022 ± 0.35 mmol/L vs. 5.62 ± 0.11 mmol/L; *p*=1.1 × 10^−5^). This significant hypoglycaemic activity was observed throughout the experiment.

Other fraction, including KLF2, KLF3, and KLF5 showed significant hypoglycaemic activity. TLC profiles indicated the presence of similar compounds in KLF2 and KLF3, which may account for the activity seen in both of these fractions (Figure 1(a)).

#### 3.1.1. Bioassay of KLF6.1–KLF6.5

OGTT of the fractions from KLF6 demonstrated that KLF6.2 and KLF6.3 showed significant hypoglycaemic activity. KLF6.2 indicated activity only in the pos-prandial regions while KLF6.3 showed activity in both the fasting (60 min) and postprandial regions (120 min) of the curve (Figure 1(b)). It is being hypothesized that the decrease in activity may be a result of the separation of compounds that worked synergistically in lowering the BGL as seen in the parent fraction, KLF6. Another reason may be due to the dose of 25 mg/kg BW that was administered, which may have been too low to produce similar efficacy as the parent fraction, KLF6 at 40 mg/kg BW.

#### 3.1.2. Bioassay of KLF6.2A–KLF6.2F and KLF6.2E-M

KLF6.2A to KLF6.2F did not show any significant hypoglycaemic activity when compared with the DMSO control (Figure 1(c)). However, KLF6.2E (dirty green solid) showed a very sharp decrease in BGL from the 90 min interval to the 150 min interval, which may indicate the presence of hypoglycaemic principle(s) whose activity was being masked by impurities (Figure 1(d)).

This fraction was recrystallized and the mother liquor (KLF6.2E-M) was bioassayed using OGTT and hypoglycaemic activity was observed from 60 minutes to 180 minutes (Figure 1(d)). The most significant hypoglycaemic activity was observed in the postprandial region and therefore may be acting in response to the glucose load and promoting the release of insulin from the beta cells. The most significant hypoglycaemic effects were observed at 90 min (4.43 ± 0.15 vs. 5.62 ± 0.11 mmol/L, *p*=0.05), 120 min (3.32 ± 0.29 mmol/L vs. DMSO group of 5.50 ± 0.17 mmol/L, *p*=0.05), 150 (3.43 ± 0.29 mmol/L vs. 4.64 ± 0.25 mmol/L, *p*=0.05), and 180 min (3.37 ± 0.89 mmol/L vs. 5.17 ± 0.34 mmol/L, *p*=0.02).

The removal of the white powdery solids resulted in hypoglycaemic activity being displayed. This indicated that this powdery solid was possibly responsible for masking the effect of the hypoglycaemic active compound(s). Purification of KLF6 to KLF6.2E increased the potency of the white crystals, hence the general decrease in hypoglycaemic activity throughout. On the other hand, further purification of the mother liquor by PLC resulted in the isolation of the active compounds responsible for the hypoglycaemic activity being observed.

**Figure 2:**
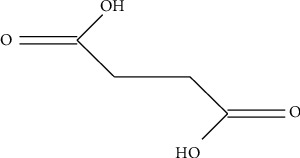
Structure of succinic acid.

The commercially available compounds were bioassayed individually (Figure 2(a)) and compared with a cocktail of the compounds (1 : 1 ratio, Figure 2(a)). The cocktail was also administered orally at 800 mg/kg BW and compared with a known hypoglycaemic agent, metformin (Figure 2(b)).

### 3.2. Gas Chromatography–Mass Spectrometer (GC-MS) Analysis Led to the Isolation of Succinic Acid and Oleic Acid

GC-MS of KLF6.2E-M-BSTFA (KLF6.2E–M derivatized with BSTFA) depicted the presence of several compounds, two of the most abundant possibly being oleic acid (OA) with a 95% match to the reference standard in the NIST library and succinic acid (SA) with an 87% match to the reference standard in the NIST library.

Analysis of the derivatized OA (OA-trimethylsilyl ester) reference standard depicted a sharp peak with a retention time of 14.08 min with the mass spectrum being displayed in the supplementary material, Figure S2. On the other hand, SA-trimethylsilyl ester reference standard gave a sharp peak at 8.93 min (Figure S1). These corresponded to two of the most intense peaks observed in the analysis of KLF6.2E-M-BSTFA. This indicated the presence of both OA and SA as two of the most abundant compounds within KLF6.2E-M (Figure S3) and may be responsible for the hypoglycaemic activity observed. The relative abundance gave a representation of the ratio of these compounds, indicating a 1 : 1.2 in favor of oleic acid. The other compounds within the fraction were in minute quantities when compared with oleic acid and succinic acid.

### 3.3. Plate-Liquid Chromatography of KLF6.2E–M

Bands 2 and 4 corresponded to the *R*_f_ values previously obtained for commercially available SA and OA reference standards, respectively. Based on the GC-MS and PLC results, Band 2 was indicated to be succinic acid while Band 4 was indicated to be oleic acid. They were further analysed using FTIR. Band 1 did not move from the origin and indicated the presence of a mixture of compounds, while the yield of Band 3 was low and was not further analysed.

### 3.4. Fourier Transformed Infrared Spectroscopy of Bands 2 and 4

**Figure 3:**
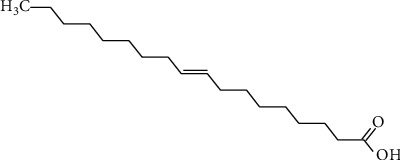
Structure of oleic acid.

Figure 3(a) indicates the peaks observed for varying wavelengths for the functional groups in Band 2. These values were compared to reference ranges of the functional groups in SA and are summarized in Table 1, where it can be seen that the experimental values corresponded with the literature values. This adds credence to the notion that Band 2 was indeed succinic acid.

Figure 3(b) indicates the various peaks obtained at different wavelengths for the functional groups present in Band 4. These values are compared to the reference range for the functional groups in oleic acid and are summarized in Table 2. It is noted that the experimental values corresponded with the literature values, indicating that Band 4 was oleic acid.

### 3.5. Synergistic and Independent Effect of SA and OA on BGL

**Figure 4:**
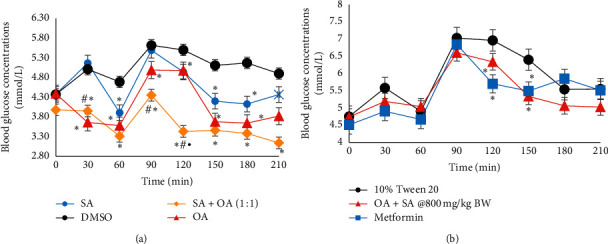
OGTT of (a) a comparison of the individually administered OA and SA versus a cocktail of OA and SA (intravenously administered at 30 mg/kg BW) and (b) a comparison of the cocktail of OA and SA (orally administered at 800 mg/kg BW) versus metformin (orally administered at 25 mg/kg BW). Data are expressed as mean ± standard error of mean, with ^*∗*^indicating statistical significance when compared with 10% DMSO control (*p* < 0.05), ^#^indicating statistical significance when compared with SA (*p* < 0.05), and ^•^indicating the statistical significance when compared with OA (*p* < 0.05).

**Figure 5:**
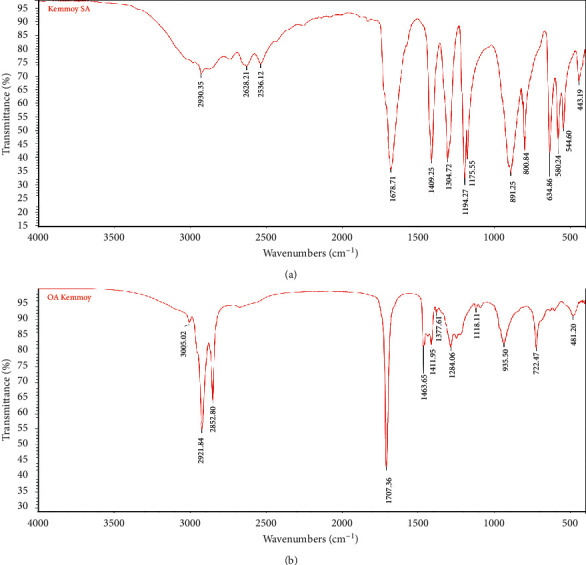
FTIR spectral analysis of (a) Band 2/succinic acid and (b) Band 4/oleic acid.

The two main compounds isolated from KLF6.2E-M, succinic acid (Figure 4) and oleic acid (Figure 5), are chiefly responsible for the hypoglycaemic activity observed throughout the experiment. Oleic acid is a naturally occurring fatty acid found in many animal and vegetable fats and oils [23]. In 2002, Obici and colleagues demonstrated that central administration of oleic acid to 10 weeks old S-D rats inhibited the production of glucose as well as improved the glucose sensitivity [23]. This was previously demonstrated in 2000 by Ryan et al. They illustrated that diets rich in oleic acid improve adipocytes glucose transport as well as insulin sensitivity in diabetic patients [24].

Succinic acid (butanedioic acid) is a dicarboxylic acid with the molecular formula of C_4_H_6_O_4._ The compound exists as a white, odourless crystalline solid with a sharp acidic taste that is found in many living organisms. Succinic acid and its alkyl esters have been proposed to be insulinotropic compounds and were suggested to be novel therapeutic approaches to type 2 diabetes [25]. The mechanism of this is believed to be done via an increase in mevalonate through the succinate thiokinase pathway, which ultimately stimulates insulin release [26]. While OA and SA have been demonstrated to have hypoglycaemic effects, no work has been done to indicate their presence in *Desmodium canum* or has shown their combined effect in glycaemic control.

The hypoglycaemic effects of the commercially available compounds when administered individually and in combination were observed (Figure 2(a)). Oleic acid showed the most significant activity and this was seen throughout the entire curve, with the most significant values seen in the postprandial region of the curve. Succinic acid also showed significant hypoglycaemic values in both the fasting and postprandial regions of the curve, with its greatest activity at the 150 min interval. Despite the difference in the activity seen between these compounds, there was a minimal significant difference between them seen only at the 30 min interval.

However, when administered as a cocktail, the hypoglycaemic effect was more potent than when administered individually. At the 30 min, 90 min, and 120 min intervals, the cocktail showed significantly hypoglycaemic activity when compared with SA at the same times (3.95 ± 0.43 mmol/L vs. 5.17 ± 0.32 mmol/L, *p*=0.0037; 4.35 ± 0.36 mmol/L vs. 5.49 ± 0.70 mmol/L, *p*=0.04 and 3.43 ± 0.22 vs. 4.94 ± 0.3, *p*=1 × 10^−5^, resp.). The cocktail also caused hypoglycaemic activity that was significantly different from OA in the postprandial at the 120 min interval (3.43 ± 0.22 mmol/L vs. 4.98 ± 0.19 mmol/L, *p*=4.29 × 10^−7^).

The increased efficacy of the drug may be a result of the blended mechanisms of action that improved the glycaemic control. In this, the succinic acid increased the release of insulin from the *β*-cells while the oleic acid inhibited the gluconeogenesis and increased the uptake into peripheral tissues. Their combined effect was especially obvious in the postprandial region, where the values fell to those in the fasting region. This is of great importance in antidiabetic therapies due to the association between postprandial hyperglycaemia and complications associated with type 2 diabetes [27].

For oral administration, the cocktail was administered orally dissolved in 10% Tween 20 which served as a better carrier than DMSO for oral administration. The cocktail showed significant hypoglycaemic effect when compared with the Tween 20 control at time intervals of 120 and 150 min (Figure 2(b)). This was compared with a known hypoglycaemic agent, metformin, which was administered at the normal dose of 25 mg/kg BW. Metformin showed significant hypoglycaemic effect at similar times of 120 and 150 min intervals. There were no statistical differences between the two groups throughout the experiment. This indicated that the naturally isolated compounds administered as a cocktail were similar in terms of efficacy as the first-line treatment of diabetes mellitus, metformin. The synergistic effect of OA and SA shows high potential in introducing a novel therapeutic approach to type 2 diabetics and should be further explored.

In conclusion, the study of the hypoglycaemic activity of the extracts of *Desmodium canum* led to the isolation of oleic acid and succinic acid from the crude ethyl acetate extract. Both compounds possess hypoglycaemic activities in the fasting and postprandial region of the OGTT. However, the synergistic effect of the compounds indicated the greatest activity which was observed throughout the entire experiment. Further studies may lead to the use of this cocktail for a therapeutic approach to diabetes mellitus.

## Figures and Tables

**Table 1:** Literature and experimental values for the various functional groups in succinic acid.

Functional group	IR reference range (cm^−1^) [22]	Experimental values (cm^−1^)
C-H stretch	2852–2962	2930.35
O-H stretch (carboxylic group)	2500–3300	Very broad peak ∼2628.21, ∼2538.12
C=O stretch	1700–1725	1678.71

**Table 2:** Literature and experimental values for the various functional groups in oleic acid.

Functional groups of oleic acid	Reference range (cm^−1^) [22]	Experimental values (cm^−1^)
C=C-H stretch	3010–3100	3005.02
R-CH=CH-R	985–1000	935.50
C-H	2850–3000	2921.84, 2852.80
C=O	1700–1725	1707.36
O-H	2500–3300	Broad peak buried in C-H stretch

## Data Availability

Data will be made available upon reasonable request.

## Abbreviations

ADA: American Diabetes Association
BG: Blood glucose
BW: Body weight
DM: Diabetes mellitus
DMSO: Dimethyl sulfoxide
EtOAc: Ethyl acetate
FTIR: Fourier Transform Infrared Spectrometry
GC-MS: Gas Chromatography–Mass Spectrometer
Hex: Hexane
KLF1–7: Fractions obtained from crude ethyl acetate extract
KLF6.1–6.5: Fractions obtained from KLF6
KLF6.2A–F: Fractions obtained from KLF6.2
KLF6.2E–M: Mother liquor obtained from recrystallization of KLF6.2E
MeOH: Methanol
BSTFA: N,O-bis(trimethylsilyl)trifluoroacetamide
OA: Oleic acid
OGTT: Oral Glucose Tolerance Test
PLC: Plate-liquid chromatography
*R*_f_: Retention factor
SA: Succinic acid
S-D: Sprague Dawley
TLC: Thin-layer chromatography
WHO: World Health Organization.

## Supplementary Materials

Supplementary Materials The supplementary document consists of the ethical approval granted by the UWI Ethics Committee to ensure ethical guidelines are followed with the use of animals within this study. This is presented in Supplementary File 1 and File 2. Additionally, the Gas Chromatography–Mass Spectrometry data of standards (oleic acid and succinic acid) and the semipurified fraction, KLF6.2-M, are also presented. Figure S1 illustrates the gas chromatogram and mass spectrum of succinic acid (butanedioic acid, >99%) trimethylsilyl ester. Figure S2 shows similar data collected from oleic acid (>99%) trimethylsilyl ester while Figure S3 shows a comparison of these standards with KLF6.2E-M. From the data, it can be deduced that both succinic acid (RT of 8.93 min) and oleic acid (RT of 14.12 min) are the two most abundant compounds present within the fraction.
