# Supplementary material for: The Isolation of Hypoglycaemic Compounds from Desmodium canum and Their Synergistic Effect on Blood Glucose Levels in Euglycaemic Sprague Dawley Rats
Source: Evid Based Complement Alternat Med. 2020 Oct 19;2020:9465139. doi: 10.1155/2020/9465139 (PMC7591975; doi:10.1155/2020/9465139)
Supplement: Supplementary Materials — The supplementary document consists of the ethical approval granted by the UWI Ethics Committee to ensure ethical guidelines are followed with the use of animals within this study. This is presented in Supplementary File 1 and File 2. Additionally, the Gas Chromatography–Mass Spectrometry data of standards (oleic acid and succinic acid) and the semipurified fraction, KLF6.2-M, are also presented. Figure S1 illustrates the gas chromatogram and mass spectrum of succinic acid (butanedioic acid, >99%) trimethylsilyl ester. Figure S2 shows similar data collected from oleic acid (>99%) trimethylsilyl ester while Figure S3 shows a comparison of these standards with KLF6.2E-M. From the data, it can be deduced that both succinic acid (RT of 8.93 min) and oleic acid (RT of 14.12 min) are the two most abundant compounds present within the fraction. [file 9465139.f1.pdf]

Supplementary Data: Ethical Approval and Gas Chromatography-Mass Spectrometry for the project entitled "*The Isolation of Hypoglycaemic Compounds from Desmodium canum and their Synergistic Effect on Blood Glucose Levels in Euglycaemic Sprague-Dawley Rats*"

Kemmoy Lattibeaudiere [1], Roy Porter [2], Ruby Lisa Alexander-Lindo[1]

[1] *Department of Basic Medical Sciences, Biochemistry Section, The University of the West Indies, Mona*

[2] *Department of Chemistry, The University of the West Indies, Mona*

Supplementary File 1: Ethical Approval Obtained in 2016 for the Project as Described in Manuscript.

THE UNIVERSITY OF THE WEST INDIES
MONA CAMPUS
Faculty of Medical Sciences
Office of the Dean

Dean: Horace Fletcher, MB, BS, DM (O&G), FRCOG, FACOG
Professor of Obstetrics and Gynaecology

May 3, 2016

Dr. Lisa Lindo
Section of Biochemistry
Department of Basic Medical Sciences
The University of the West Indies
Mona, Kingston 7

Dear Dr. Lisa Lindo,

Re: Kemmoy Lattibeaudiere research proposal entitled- Investigating the effects of various plant extracts on testosterone levels, blood glucose concentration, blood pressure and penile erection in Sprague-Dawley rats. AN 07, 15/16

Thank you for submitting the above mentioned proposal for review by the UWI Ethics Committee.

The proposal was reviewed and approved, having met the required ethical standards. The extended approval period commences on May 4, 2016 and will end on May 3, 2017. However, as per the intention of the principal investigator to conduct the same study using 35 additional plants listed in the proposal, Dr. Lindo should be requested to submit to the Ethics committee the following:

1. A summary page outlining the specific plant selected at each phase of the study

2. Name(s) of student(s) assigned to carry out the additional investigations on the other plant species

Mona, Kingston 7, Jamaica W.I.

Tel: (876) 927-1297, (876) 927-2556
Fax: (876) 977-2289, (876) 977-3470
Email: horace.fletcher@uwimona.edu.jm

3. A completed "Animal Checklist" for the use of additional Sprague-Dawley rats to conduct further investigations using any of the other plants listed in this AN7 15/16

Yours sincerely,

Professor Horace Fletcher
Chairman UWI Ethics Committee

Supplementary File 2: Ethical Renewal Granted in 2017 for the Continuation of the Project as Described in the Manuscript

THE UNIVERSITY OF THE WEST INDIES
MONA CAMPUS
Faculty of Medical Sciences
Office of the Dean

Dean: Horace Fletcher, MB, BS, DM (O&G), FRCOG, FACOG
Professor of Obstetrics and Gynaecology

May 11, 2017

Dr. Lisa Lindo
Section of Biochemistry
Department of Basic Medical Sciences
The University of the West Indies
Mona, Kingston 7

Dear Dr. Lisa Lindo,

Re: Kemmoy Lattibeaudiere research proposal entitled- Investigating the effects of various plant extracts on testosterone levels, blood glucose concentration, blood pressure and penile erection in Sprague-Dawley rats. AN 07, 15/16

Thank you for submitting the above mentioned proposal for review by the UWI Ethics Committee.

The proposal was reviewed and approved, having met the required ethical standards. The extended approval period commences on May 11, 2017 and will end on May 12, 2018.

Yours sincerely,

Professor Horace Fletcher
Chairman UWI Ethics Committee

Mona, Kingston 7, Jamaica W.I.
Tel: (876) 927-1297; (876) 927 2556; (876) 927-1660-9 **Email:** horace.fletcher@uwimona.edu.jm
Fax: (876) 977-2289; (876) 977-3470

The Gas Chromatography-Mass spectrometric analysis of the semipurified fraction, KLF6.2E-M was done as described in text, section 2.2.3. The fraction was compared to butanedioic acid (succinic acid; >99% purchased from Sigma) and oleic acid (>99% purchased from Sigma). Figure S1 shows the analysis of the succinic acid standard derivitized with N,O-bis (trimethylsilyl)trifluoroacetamide (BTSFA). A retention time (RT) of 8.90 min was observed from the GC analysis. Mass spectral analysis of the compound shows varying fragments with the most abundant peak being 147 m/z followed by 73 m/z. The varying fragments presented a molecular ‘fingerprint’ for said compound.

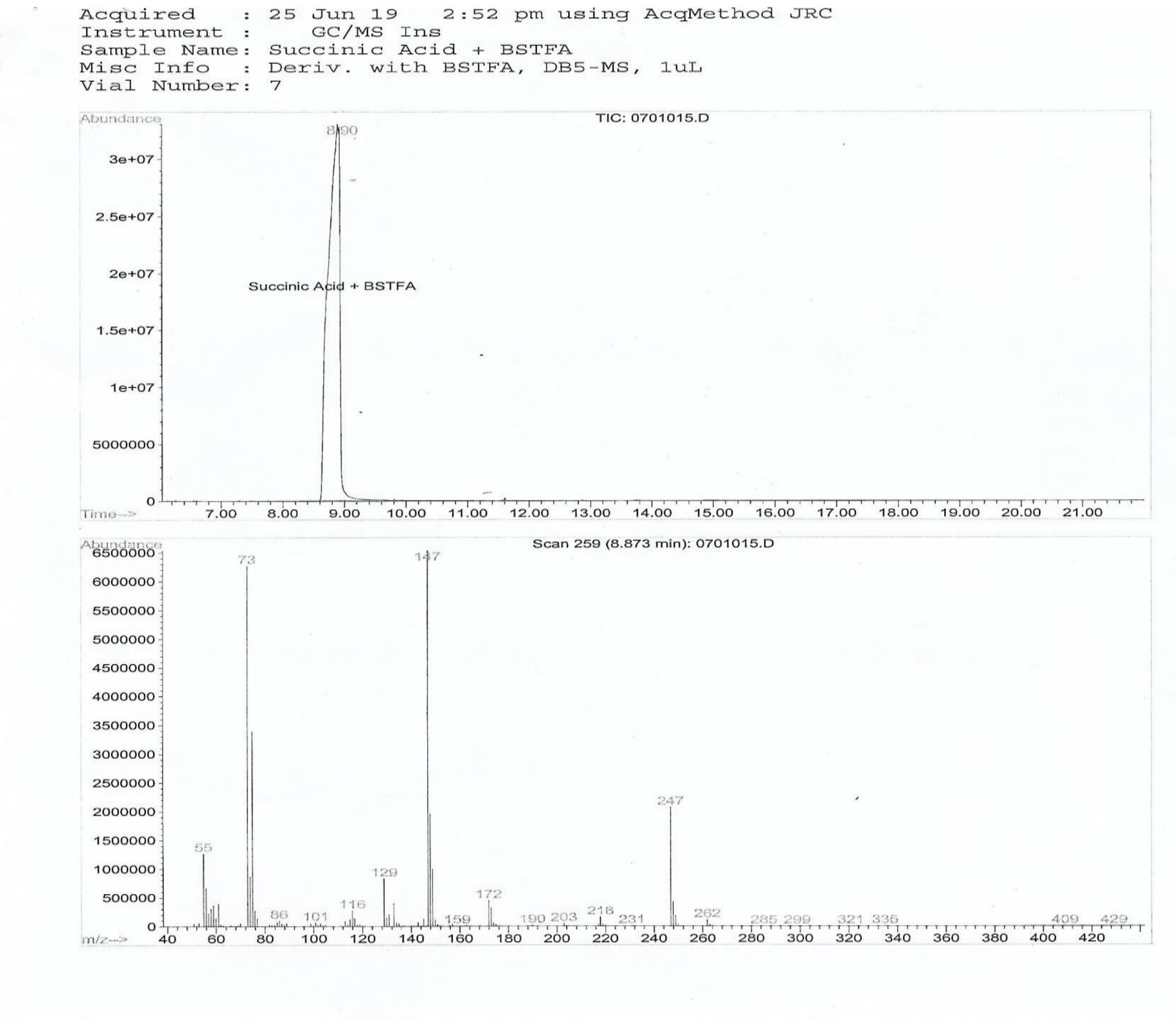

Figure S1: Gas Chromatography-Mass spectrum analysis of Butanedioic acid, bis (trimethylsilyl) ester

A silmilar analysis of oleic acid-BSTFA was done and displayed a RT of 14.08 min. Mass spectrum displayed the fragments with the most abundant peak being 73 m/z (Figure S2). The mass spectrum once again provides a ‘fingerprint’ for oleic acid which is then compared to the sample, KLF6.2E-M to confirm the presence of oleic acid. The data can be found in Figure S3

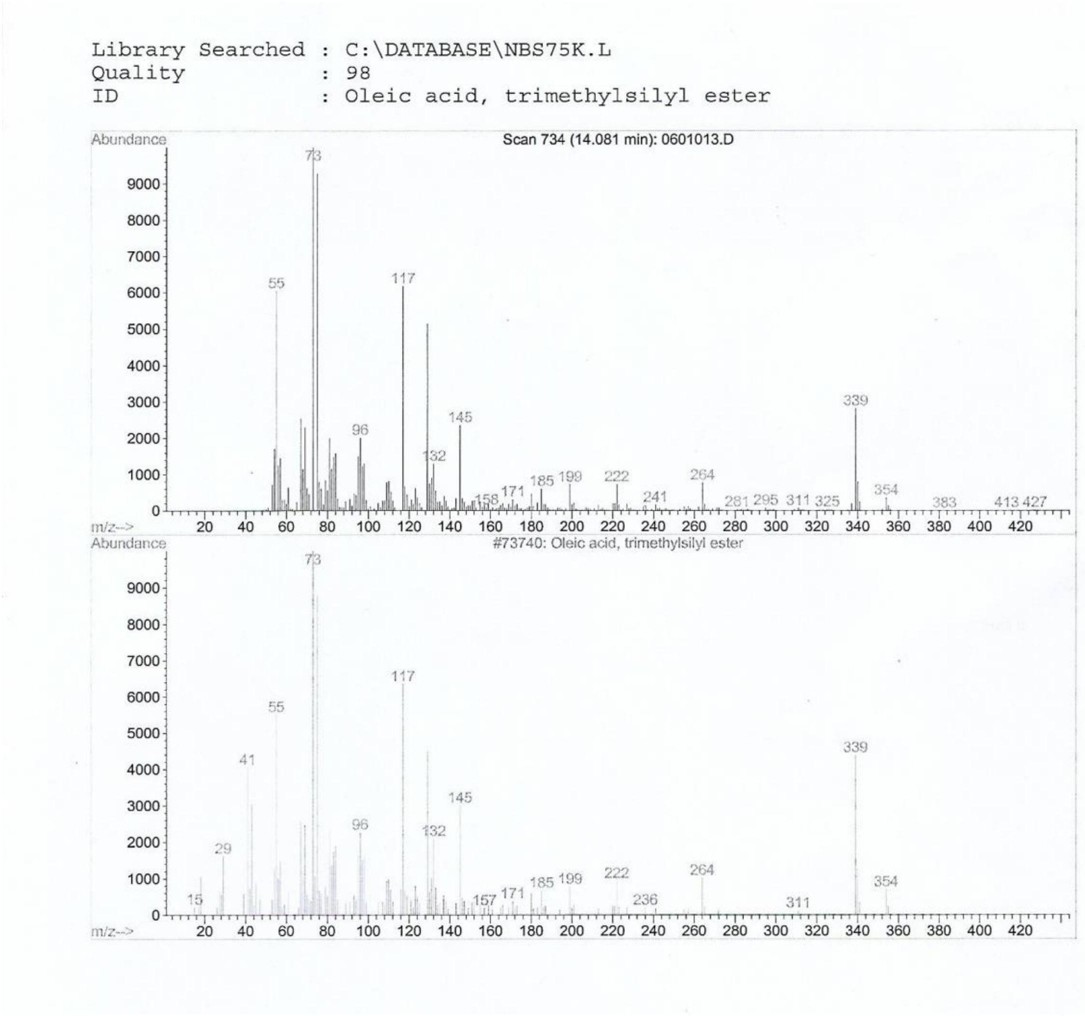

Figure S2: Mass spectrum analysis of Oleic acid, bis (trimethyl silyl) ester

A comparison to KLF6.2E-M derivitized with BSTFA was made with these standards, where it was observed that these compounds. It was observed that both succinic acid (RT of 8.93 min) and oleic acid (RT of 14.12 min) were the two most abundant compounds present within the fraction. Other compounds were also observed in lower quantity, however, succinic acid and oleic acid are believed to be synergistically responsible for the hypoglycaemic activity documented.

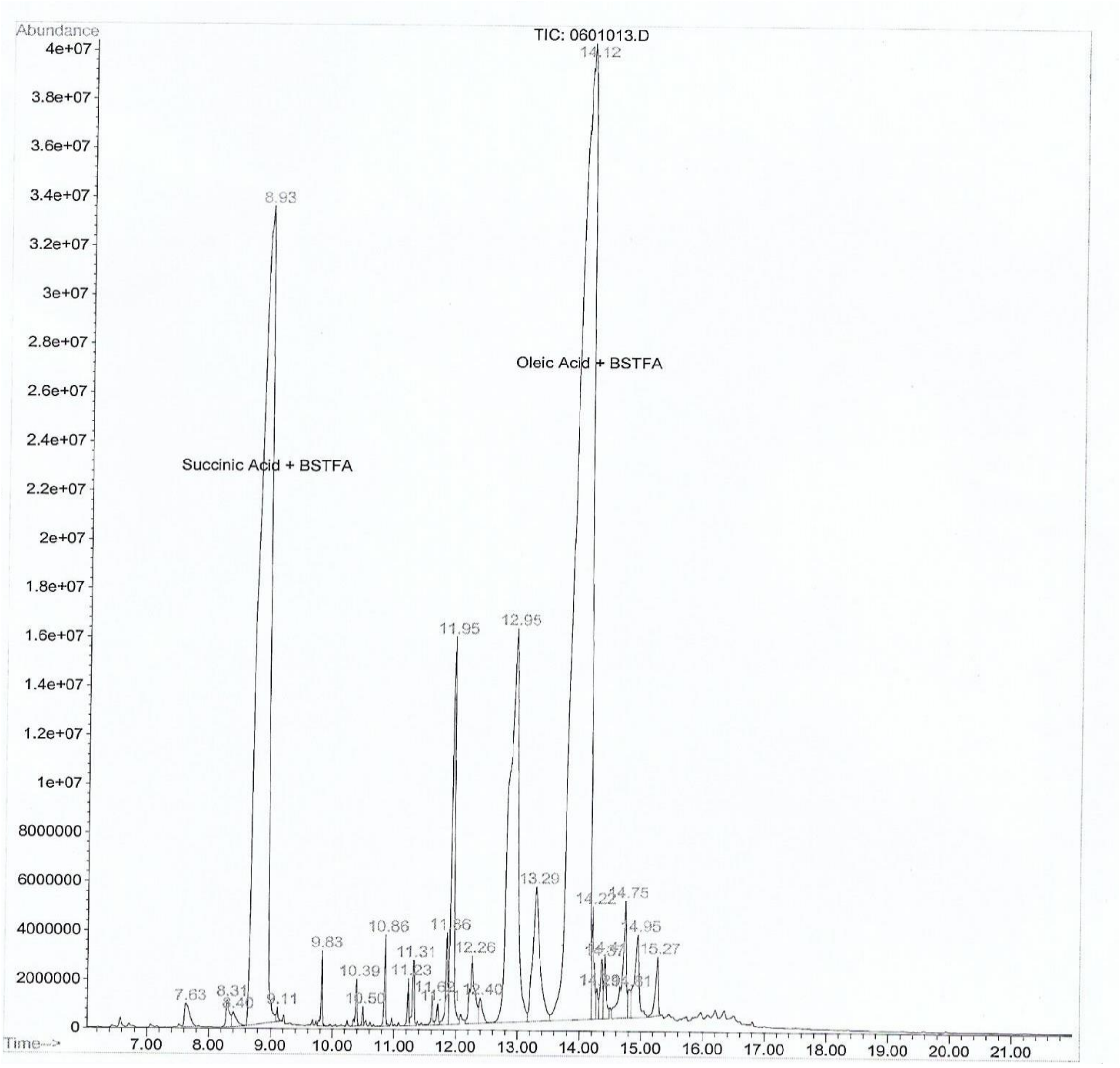

Figure S3: Gas chromatography-Mass Spectrum analysis of KLF6.2E-M indicating the presence of succinic acid and oleic acid
